# A hyperfine-resolved spectroscopic model for vanadium monoxide (^51^V^16^O)

**DOI:** 10.1080/00268976.2023.2255299

**Published:** 2023-09-06

**Authors:** Charles A. Bowesman, Sergei N. Yurchenko, Jonathan Tennyson

**Affiliations:** Department of Physics and Astronomy, University College London, London, UK

**Keywords:** Hyperfine coupling, variational nuclear motion, spectroscopy, potential energy curves, vanadium monoxide

## Abstract

Vanadium monoxide ( 51V 16O) is believed to play an important role in the atmospheres of hot-Jupiters, but high-resolution studies have so far failed to detect it, at least in part because of the inaccuracy of available lists. It is likely that the large hyperfine splittings in the spectra of VO, arising from the large nuclear spin $I=\frac{7}{2}$ of the 51V atom, has contributed to the non-detections with the current hyperfine-unresolved VOMYT line list. To aid in the production of a new line list, a fully hyperfine-resolved spectroscopic model has been constructed which includes 15 low-lying electronic states (6 quartets and 9 doublets) of VO with the inclusion of hyperfine couplings based on use of the new, hyperfine-resolved version of the diatomic variational nuclear motion programme Duo. The new spectroscopic model is refined against empirical Marvel energies derived from experimental transitions, and hyperfine couplings are fit for the 3 electronic states for which hyperfine effects have been resolved in lab spectra. This model is used to assign some previously identified perturbations.

## GRAPHICAL ABSTRACT

**Figure:**
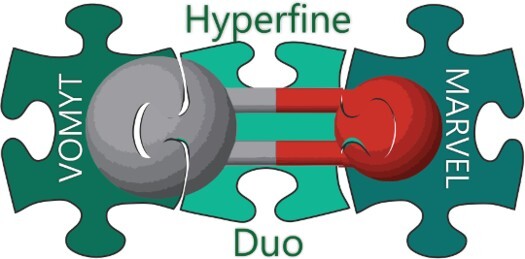

## 1. Introduction

Vanadium monoxide ( 51V 16O) is a well-studied open shell diatomic molecule. The electronic structure of VO has long been the subject of research, with many early investigations aiming to characterise the X4Σ− ground state [1–3] and subsequent work focussing on its wealth of low-lying excited states [4–9]. As with other 3d transition metal oxides, the complex electronic structure of VO gives rise to complicated and dense spectra [8,10–14]. For VO, these complexities are the result of the three open-shell electrons, arising from the 23 electrons of Vanadium, five of which are unpaired, split over seven orbitals and the ( 3dσ+3dπ) bond with Oxygen of triple bond character [5,15–17]. The problem is further complicated both by the large number of strong resonances between the densely packed electronic states [11,18–24] and the need to consider hyperfine-resolved spectra as 51V has nuclear spin $I=\frac{7}{2}$ which leads to a large hyperfine splittings in a number of key states.

While polyatomic vanadium oxides have been analysed for their material properties [25,26], with many forms being semiconductors [27], VO occurs primarily in the gas phase. It has frequently been studied in an astrophysical context, where it is known to exist in the atmospheres of cool dwarf stars [28–40] and Mira variables [41–43]. It has also been of interest in the atmospheres of exoplanets such as hot and ultra-hot Jupiters [44], where a recent study claimed to detect it in the atmosphere of WASP-76b using high-resolution cross-correlation spectroscopy [45]. This detection used the existing, hyperfine-unresolved ExoMol line list of McKemmish *et al.* named VOMYT [46] which was computed based on empirically-determined Morse-oscillator potentials for most states and *ab initio* coupling curves from MRCI calculations [17]. This comes after years of non-detections, often citing the inaccuracies of existing hyperfine-unresolved line lists as the cause [47]. Accordingly, it would be of use to the astrophysical community to produce a hyperfine-resolved line list for high-resolution studies.

Perturbation theory-based effective Hamiltonians have been extensively used to represent the energy levels of VO [19–24,48–53]. However, given the large number of observed perturbations in the spectra of VO and the large number of electronic state crossing seen in *ab initio* potential calculations, effective Hamiltonian calculations are unlikely to adequately characterise the perturbations and resonances [54]. Increasingly people have been developing spectroscopic models for open shell diatomic molecules based on the use of potential energy curves (PECs) and appropriate couplings. Examples include *ab initio* studies of the hyperfine-resolved spectrum of H 2 [55], the work of Augustovicova and Spirko [56,57], Havalyova *et al.* [58] and the MOLLIST project [59], which employed a hybrid approach, as well as our own ExoMol project [60]. For example, our study of the lowest 5 electronic states of CaO using a variational model [61] led to the reassignment of the vibronic states for a large portion of the experimentally derived energies, extending these assignments to much higher *J* values and proposing a new description of a number of the observed resonances.

In this work we present a comprehensive model of 15 low-lying electronic states of VO; VO is a considerably more complicated system than CaO and to get to this stage we have had to undertake a number of preparatory steps. Bowesman *et al.* [24] performed a MARVEL (measured active rotation-vibration energy levels) study for rovibronic transitions between 13 of the 15 electronic states considered here, obtaining 4 402 hyperfine-resolved energy levels and a further 4 712 hyperfine-unresolved levels. This study also assigned lines in the 2 2Π – X 4Σ− band system allowing levels in the quartet and doublet state to be put on a single energy scale. The earlier marvel results are extended here with the newly available experimental results of Dörring *et al.* [62]. Qu *et al.* [63] developed a fully hyperfine-resolved version of our workhorse variational diatomic nuclear motion programme Duo [64]; they used this programme to develop a hyperfine-resolved model for the VO electronic ground state [65], which we expand upon here to include additional hyperfine-resolved electronic states. Qu *et al.* [66] also developed a preliminary, hyperfine-unresolved, 11 electronic state model for VO. These steps all provide essential input for the present study.

The paper is organised as follows. Section 2 outlines the implementation of the spectroscopic model and the fitting process. Section 3 describes the quality of the new model and details the assignment of some previously unassigned experimentally observed electronic perturbations. Section 4 presents some discussion on *ab initio* works on the electronic structure of VO. Section 5 gives our conclusions and an overview of future work to follow.

## 2. Spectroscopic model

To construct an initial form for the new model, the 11 potential energy curves (PECs) for the X 4Σ−, A ′4Φ, A 4Π, B 4Π, C 4Σ−, D 4Δ, 1 2Δ, 1 2Σ+, 1 2Φ, 1 2Π and 2 2Π states were taken from Qu *et al.* [66] and those for the 1 2Γ and 1 2Σ− states from McKemmish *et al.* [46]. PECs for the 2 2Δ and 3 2Δ states were added based on the fitted effective Hamiltonian parameters given by Bowesman *et al.* [24]. Most of the *ab initio* diagonal and off-diagonal spin-orbit and Lx curves calculated by McKemmish *et al.* [17] were included. For the curves that had been refit by Qu *et al.* [66], the updated versions were included instead. This includes all of the spin-rotation and spin-spin curves. The hyperfine coupling curves calculated by Qu *et al.* [65] for the X 4Σ− were also used. The dissociation energy of VO has been experimentally measured using a variety of techniques [16,67–71] but here we adopt the value of 52790cm−1 determined by Merriles *et al.* [72]; since all the electronic states we consider go the same limit this value is used to constrain the asymptotes of all states.

It should be noted that while the 11 electronic state model of Qu *et al.* [66] gave a good description of the term energies, the model only included two spin-orbit couplings. Given that spin-orbit coupling facilitates intensity borrowing between coupled states, a model with a more complete description of the spin-orbit couplings between the states of VO was necessary for our ultimate goal of constructing a new line list.

### 2.1. Duo model

The model was computed using the programme Duo [73] over a grid of 4001 points arranged between 1.2 and 4.0 Å. The rotational basis extends up to *J* = 153.5, so as to compute levels up to *F* = 150. An energy cutoff was imposed at 45000cm−1 to make the calculation feasible and the vibrational basis size for each electronic state was set accordingly and are given in Table 1. This is the same energy cutoff used in the calculation of the existing VOMYT line list [46]. At high energies close to dissociation the density of states increases significantly as sequential vibrational states interact with one another. In order to ensure the vibrational levels below the energy cutoff had accurate energies, each state's vibrational basis was set to extended beyond the energy cutoff.

**Table 1.** The potential minimum, Te, and equilibrium bond length, re, of all 15 states included in the spectroscopic model. These values are rounded to 3 decimal places; the full values are available in the Duo input file included with the supplementary material. The vibrational basis size for each state, vmax, and the maximum value of *v* for each state that occurs below the energy cutoff of 45000cm−1, vcutoff, are also provided.

State	Te (cm −1)	re (Å)	vmax	vcutoff
X 4Σ−	0.000	1.589	75	66
1 2Σ−	5604.265	1.574	65	56
A ′4Φ	7289.736	1.625	65	55
1 2Γ	8503.438	1.577	64	50
A 4Π	9529.978	1.635	65	52
1 2Δ	9861.072	1.581	64	53
1 2Σ+	10392.666	1.590	64	54
B 4Π	12656.367	1.641	60	49
1 2Φ	15438.229	1.627	54	40
1 2Π	17116.891	1.630	54	39
C 4Σ−	17492.985	1.672	55	43
2 2Π	18106.524	1.623	54	47
D 4Δ	19241.935	1.686	45	26
2 2Δ	25093.236	1.677	44	30
3 2Δ	31976.453	1.672	35	21

The model was refined against the empirical energy levels produced from a marvel analysis of the published VO transition data [24,74]. This marvel data set consists of 5 702 hyperfine-resolved and 4 712 hyperfine-unresolved energy levels, derived from experimental transitions from 15 sources; 14 sources were initially compiled by Bowesman *et al.* [18–23,48,50–53,75–77] with an additional 1 439 hyperfine-resolved transitions of Döring *et al.* [62] added in a recent update [74]. Refinement was initially carried out in two stages: first the potentials and couplings were fit against only the hyperfine-unresolved marvel energy levels. All of the 15 electronic state potentials, 3 spin-rotation and 6 spin-spin coupling curves were refined at this stage. 10 of the 11 diagonal spin-orbit coupling curves were refined; the diagonal spin-orbit coupling of the 1 2Γ state was left with its original *ab initio* form due to a lack of observational data to fit against. 18 of the 28 off-diagonal spin-orbit coupling curves were also refined, including all of those that had been fit by Qu *et al.* [65,66].

The 15 PECs were represented using analytical forms, 14 of which were represented by extended Morse oscillators (EMO) [78–80] of the form

(1) $$\begin{array}{rl} V\left(r\right) & =Te+\left(Ae-Te\right)\\ & \;\times \left[1-\exp\left(-\sum_{i=0}^{N}Bi\xi_{p}^{i}\left(r-re\right)\right)\right]2, \end{array}$$

where Ae−Te=De is the dissociation energy, Ae is the corresponding asymptote, Te is the potential minimum, re is the equilibrium distance of the PEC, and ξp is the Šurkus variable [81] given by

(2) $$\xi p=\frac{rp-r_{e}^{p}}{rp+r_{e}^{p}}.$$

All of the PECs in this model represented using the form given by Equation (1) have *N* = 2 or 3. The PEC of the 1 2Γ state was represented with a standard Morse oscillator, due to a lack of experimental data for this state to fit against. The Te and re values used for each state are shown in Table 1; the individual *p* values and Bi parameters are given in the Duo input file provided as supplementary material.

**Figure 1.**
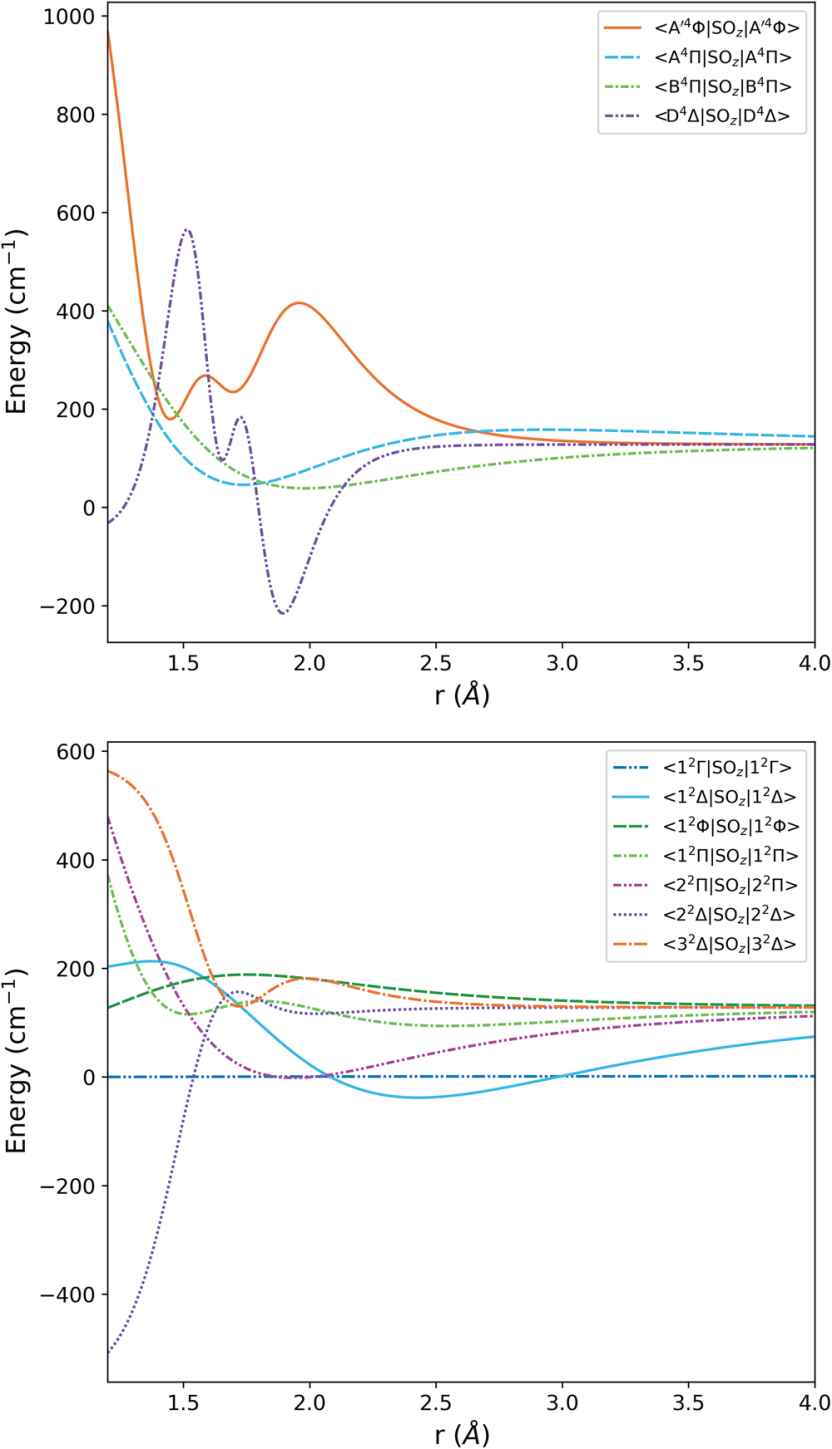
The diagonal spin-orbit coupling curves of 51V 16O included in this model. The upper plot shows the spin-orbit coupling curves for the quartet states and the lower plot the curves for the doublet.

**Figure 2.**
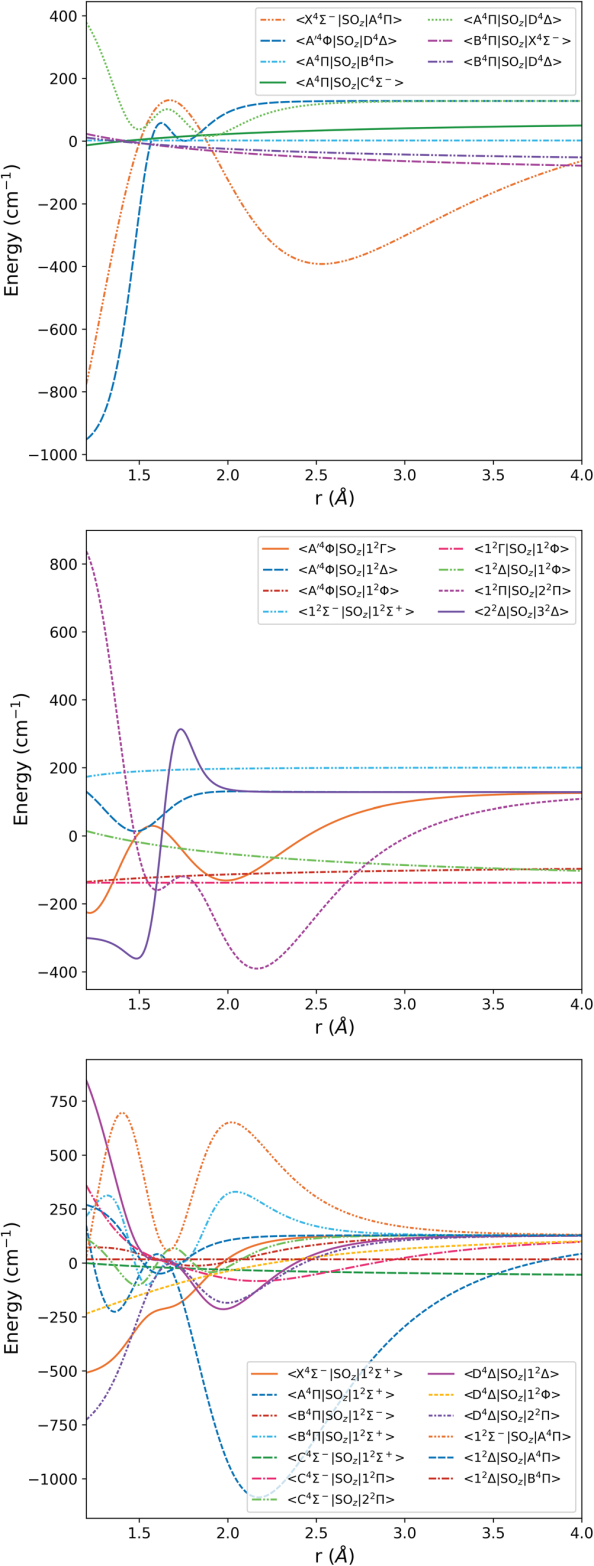
The off-diagonal spin-orbit coupling curves of 51V 16O included in this model. The upper plot shows the spin-orbit couplings between different quartet states, the middle plot those between different doublet states and the bottom plot those between a quartet and a doublet state.

The spin-orbit coupling curves presented by Qu *et al.* [66] were represented using polynomials of the form

(3) $$V\left(r\right)=\sum_{i=0}^{N}ai\left(r-re\right)i$$

while those of McKemmish *et al.* [17,46] were a series of discreet *ab initio* grid points. To refine these spin-orbit coupling curves, all of the *ab initio* forms were fit to analytical representations. While the polynomial representations of Equation (3) are adequate when fitting to a wide range of data, the VO marvel data generally occupies low vibrational states. This means that the experimentally known states primarily reside low-down in their respective potentials. Consequently, while our fits are well defined at values of *r* close to the potential minimums at their respective re values, they poorly constrained towards the edges of our calculation and lead to over-fitting. This was especially true towards the 4.0 Å end of our grid, where the polynomial representations of the spin-orbit couplings had a tendency to extrapolate to extremely high values. As we aim to produce a line list up to close to dissociation, we require analytical representations that did not produce unrealistically large magnitudes for the spin-orbit couplings at large or small *r*. To achieve this, we instead fit spin-orbit couplings to a functional form suggested by Le Roy [82]

(4) $$F\left(r\right)=\left(1-\xi p\right)\sum_{k=0}^{N}Bk\xi_{p}^{k}+\xi pB\infty ,$$

where B∞ is an asymptote reached as r→∞. By constraining the value of this asymptote it was easy to avoid the spin-orbit couplings which extrapolate to extreme values. All of the refined spin-orbit coupling curves were represented with the form given in Equation (4), as well as one of the spin-rotation and one of the spin-spin coupling curves. The diagonal spin-orbit coupling curves are shown in Figure 1 and the off-diagonal curves in Figure 2. Several of the curves shown in Figure 2 appear almost flat; these curves are the unchanged *ab initio* curves of [17]. Though the refined spin-orbit couplings are in many cases considerably changed from their initial *ab initio* forms, the resultant model reproduces empirical term energies much more accurately.

While the use of the analytic representation given by Equation (4) was helpful in avoiding extrapolation to high magnitude spin-orbit couplings at large-*r*, some couplings still tend to large values on the order of hundreds of wavenumbers at the low-*r* limit. While these are likely not representative of the true magnitudes of couplings at those bond lengths, the low-*r* configurations generally correspond to positions in the respective potentials with very high energies, sometimes above the dissociation limit. As such, these few couplings with doubtful low-*r* extrapolation should not impact the overall quality of the model. Indeed, these couplings are of these forms as their values closer to the equilibrium bond length were crucial in obtaining a good fit to experimental data.

### 2.2. Hyperfine couplings

Seven hyperfine coupling terms are implemented in Duo [63] and have been used to produce an empirical, hyperfine-resolved model for the X 4Σ− ground state of VO [65]. As is standard in the formalism of Duo, these couplings are implemented as radial functions of the bond length *r* (Å) and can be constructed using a variety of analytical representations [73].

The Fermi contact parameter bF describes the interaction between the electron spin and nuclear spin and has magnitude proportional to the electron spin density at the position of the atomic nucleus. Accordingly, this parameter is larger for states arising from configurations with an unpaired electron occupying an *s* orbital. While the Fermi contact interaction is usually the largest contributor to hyperfine-splitting, the diagonal and off-diagonal by 2 nuclear electric quadrupole interactions, eQq0 and eQq2, arising from a distortion of the nuclear electric charge distribution, are also significant when the nuclear spin is large ( $I>\frac{1}{2}$) [83]. Other hyperfine couplings considered are the nuclear spin-orbit interaction *a*, the electron spin nuclear spin dipole-dipole interactions *c* and *d*, and the nuclear spin-rotation interaction ci: their effects and implementation are described by Qu *et al.* [63].

After a satisfactory fit was achieved to the hyperfine-unresolved data, determined based on the global root-mean-square error (RMSE), hyperfine couplings were added to the model. Refinement was then carried out against the 5 702 hyperfine-resolved marvel energy levels, which only cover the X 4Σ−, B 4Π, C 4Σ− and 1 2Σ+ states. While this stage primarily involved fitting of the various hyperfine coupling curves, small changes were also made to the electronic state potentials, spin-rotation and spin-spin coupling curves of these states.

Initial forms for the hyperfine coupling curves of the X 4Σ− state were taken from the ground state hyperfine-resolved model of Qu *et al.* [65], which consisted of Fermi contact, nuclear electric quadrupole, electron spin – nuclear spin dipole-dipole (*c*) and nuclear spin-rotation coupling curves. For the hyperfine couplings of the B 4Π and C 4Σ− states, initial values for the couplings were set based on the values obtained via effective Hamiltonian fits to hyperfine-resolved experiments [19,22]. Both of these states were fit with all of the same class of couplings as the X 4Σ− state, except for the B 4Π state which was fit with additional nuclear spin-orbit (*a*) and electron spin – nuclear spin dipole-dipole (*d*) couplings as they were deemed necessary in the experimental fits [22].

**Figure 3.**
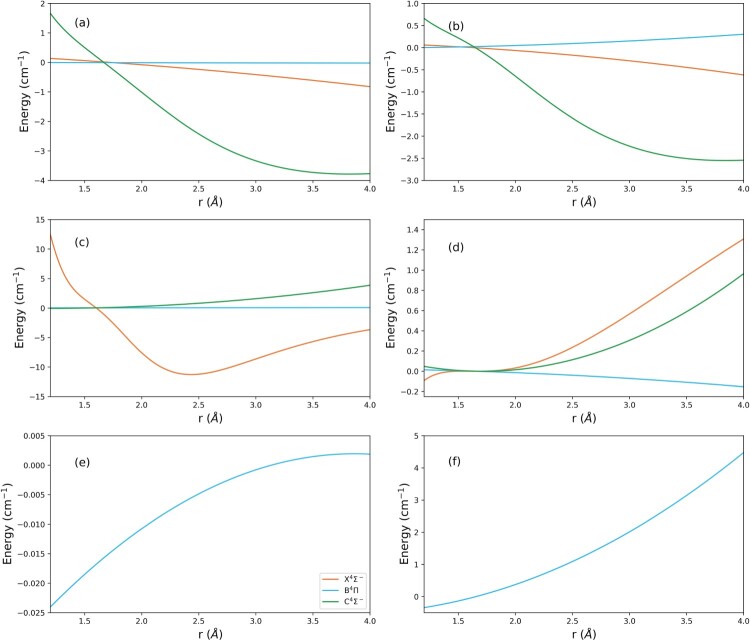
The six hyperfine coupling curves included in the new VO spectroscopic model: (a) Fermi contact parameter, bF; (b) Diagonal nuclear electric quadrupole interaction, eQq0; (c) Nuclear spin-orbit interaction, *a*; (d) Nuclear spin-rotation interaction, ci; (e) Electron spin nuclear spin dipole-dipole interaction, *c*; (f) Electron spin nuclear spin dipole-dipole interaction, *d*.

The initial forms for all of the hyperfine couplings were polynomial representations described by Equation (3). As with other couplings in the model, it was again necessary in some cases to instead use the representation given by Equation (4) to avoid unreasonable extrapolation at large-*r*. The final forms of each coupling used in this model are shown in Figure 3. Though we expect the Fermi contact parameter to be the dominant hyperfine coupling parameter for the ground state, it was necessary to fit a much larger nuclear electric quadrupole interaction to reproduce experimental energies.

The programme Duo was developed in part to fit model parameters to hyperfine-unresolved energies, but the fitting of hyperfine coupling parameters is a new addition. The finalisation of this new hyperfine refinement module is forthcoming and will be made available via GitHub when complete.

## 3. Results

Though VO is a diatomic molecule, the inclusion of a large number of electronic states and a multitude of couplings result in a fairly complex Hamiltonian which, when combined with the expansion of the basis set under hyperfine coupling, requires a considerable amount of computation power to solve. Hence, the final model required 1.3 TB of high-performance RAM to compute the 3 410 598 states present below the 45000cm−1 cutoff. The final model contains three states with full hyperfine resolution, the X 4Σ−, B 4Π and C 4Σ− states, and 12 states without, the A ′4Φ, A 4Π, 1 2Δ, 1 2Σ+, 1 2Φ, 1 2Π, 2 2Π, D 4Δ, 2 2Δ and 3 2Δ states. While the 1 2Σ+ has observed hyperfine-splitting through its perturbations to the B 4Π state [21,22], the coverage of this 1 2Σ+ is sparse and insufficient to meaningfully fit any hyperfine couplings to. Hence in the 12 states without hyperfine couplings, any apparent hyperfine splitting is induced through coupling to the X 4Σ−, B 4Π or C 4Σ− states.

Table 2 shows the obs.-calc. energy root-mean-square error (RMSE) for the newly computed hyperfine-resolved Duo model, calculated against the known hyperfine-resolved marvel levels. Table 3 gives the same comparison for all of the hyperfine-unresolved marvel levels. In this comparison, hyperfine-unresolved marvel levels are compared against all of their equivalent hyperfine components. This also includes levels from the X 4Σ−, B 4Π and C 4Σ− states with either higher *F* or *v* quantum numbers than those covered by the hyperfine-resolved data. Consequently, the RMSE of hyperfine-unresolved data for the X 4Σ−, B 4Π and C 4Σ− states shown in Table 3 is noticeably worse than that of the hyperfine-resolved data in Table 2. The high RMSE values of the hyperfine-unresolved C 4Σ−, *v* = 1, 2 and 2 2Π, *v* = 0 data are the result of a small number of poorly fit levels where these states perturb one another.

**Table 2.** Observed minus calculated RMSE for the experimentally derived, hyperfine-resolved MARVEL energies and their values calculated using Duo, for each vibronic state.

State	*v*	F range	RMSE (cm −1)	No. energies
X 4Σ−	0	0 – 47	0.060	1 270
	1	0 – 36	0.150	865
	2	5 – 34	0.183	413
1 2Σ+	2	35 – 43	0.819	13
B 4Π	0	1 – 46	0.229	1 841
C 4Σ−	0	1 – 48	0.126	1 287
Overall			0.169	5 689

**Table 3.** Observed minus calculated RMSE between the experimentally derived, hyperfine-unresolved MARVEL energies and the energies of their equivalent hyperfine components calculated using Duo, for each vibronic state.

State	*v*	F range	RMSE (cm −1)	No. energies
X 4Σ−	0	48 – 83	0.244	832
	1	37 – 83	0.282	940
	2	35 – 71	0.232	520
A ′4Φ	0	0 – 55	0.234	2 240
	1	1 – 51	0.420	1 600
	2	10 – 46	0.337	848
A 4Π	0	0 – 82	0.276	4 316
1 2Δ	0	0 – 50	0.200	1 320
	1	5 – 51	0.190	640
1 2Σ+	2	47 – 61	0.272	24
	3	24 – 33	0.117	16
B 4Π	0	47 – 82	0.443	744
	1	0 – 46	0.351	2 080
1 2Φ	0	1 – 50	0.061	1 296
1 2Π	0	1 – 50	0.121	1 296
	1	0 – 50	0.135	1 168
	2	5 – 46	0.185	552
	3	5 – 39	0.147	440
C 4Σ−	0	49 – 84	0.737	518
	1	9 – 83	0.811	1 062
	2	11 – 76	1.242	739
2 2Π	0	0 – 68	1.355	900
	1	0 – 33	0.451	240
D 4Δ	0	0 – 55	0.649	2 460
	1	1 – 45	0.450	1 592
2 2Δ	0	0 – 48	0.126	1 264
3 2Δ	0	3 – 50	0.081	848
Global			0.470	30 495

It is notable that, excluding the limited data on the 1 2Σ+ state, none of the doublets have observed hyperfine-splittings. This is perhaps not surprising however, as some observations of the doublet states in the 1 2Π–1 2Δ and 2 2Π–1 2Δ bands [20] and all of the published observations of the 2 2Δ–1 2Φ, 1 2Φ–1 2Δ and 3 2Δ–1 2Δ bands [20,51,52] were unable to resolve Λ-doubling.

Duo assigns quantum number labels to the hyperfine-resolved states it calculates under Hund's case (a β) coupling. Due to the complicated number of spin-orbit couplings present in the model, the labelling of Ω values is sometimes wrong. This can be corrected however, through manual inspection of plots of the calculated energy as a function of *F* for each vibronic band. While this problem also occurs without hyperfine-coupling, a new issue is the assignment of the label *J*, which is not rigorous under hyperfine coupling. Accordingly, in cases such as the internal hyperfine perturbations of the X 4Σ− state observed in the literature [19,22,48,53,84], *J* is sometimes mislabelled by Duo but can be easily corrected in post-processing.

It should be noted that a smaller vibrational basis size was used when fitting this model. This is because roughly twenty thousand fitting iterations were necessary to fit the high correlated parameters of the model, so a reduced basis size allowing for the calculation of term energies within approximately 15 minutes was used. A series of final fits were done to the ground state hyperfine coupling curves using the full ground state vibrational basis size in order to ensure that the fit was converged, taking approximately one hour per iteration. In contrast, one iteration with the final vibrational basis size used in the complete model takes 12 hours to compute. The final fits to the ground state hyperfine coupling curves were essential to ensure that the quality of our fit was retained for the final model. Our final model, in the form of an input file for programme Duo, is given as supporting information to this paper.

### 3.1. Perturbations

Perturbations in the spectra of VO have been observed across multiple bands; these are summarised by us previously [24]. Perturbations to the B 4Π, v=0,1–X 4Σ− bands by the 1 2Σ+, *v* = 2, 3 states were identified by Merer and co-workers [21,22] and comprise the only experimental knowledge of the 1 2Σ+ state. Perturbations to the C 4Σ−, v=0,1,2 states observed in C 4Σ−–X 4Σ− bands by Lagerqvist and Selin [18] and Cheung *et al.* [19] have remained unassigned, however. We now assign the perturbations to the C 4Σ−, *v* = 1 (F 2) state at *J* = 50.5−51.5 to the 2 2Π3/2, *v* = 0 state, as well as those seen in the C 4Σ−, *v* = 1 (F 3) state at *J* = 62.5−64.5. Similarly, the perturbations to the C 4Σ−, *v* = 2 (F 3) state at J=25.5−27.5 arise from the 2 2Π3/2, *v* = 1 state. These new assignments are provided in the marvel input file given in the supplementary material. The perturbations to C 4Σ−, *v* = 0 at *J* = 24.5, 38.5, 73.5−76.5 remain unidentified. The electronic perturbation to the D 4Δ–A ′4Φ (0, 0) band observed by Merer *et al.* [20] likewise remains unassigned. Further experimental measurements targeting the as of yet unobserved electronic states of VO would likely enable the identification of these perturbing states.

## 4. Discussion

The model presented here is extensive in its coverage of 15 electronic states; however, *ab initio* electronic structure calculations indicate that there are a significant number of additional electronic states that we do not observe. Hübner *et al.* [15] calculate a further 9 doublet states between 21 000 – 28000cm−1, below the observed 3 2Δ state. They also report 4 additional quartet states between 31 000 – 45 000 cm −1 and 6 sextet states between 24 000 – 32 000 cm −1. Indeed, according to Wigner-Witmer correlation [83] each of the two dissociation channels below the VO ionisation energy of 58 380 cm −1 [85] should correlate to 36 electronic states: the O( 3P) + V(3d 34s 2, 4F) asymptote to two Σ+, one Σ−, three Π, three Δ, two Φ and one Γ state each of doublet, quartet and sextet multiplicity; the O( 3P) + V(3d 44s 1, 6D) asymptote to one Σ+, two Σ−, three Π, two Δ and one Φ state each of doublet, quartet, sextet and octet multiplicity. It is likely that the higher dissociation channels will also give rise to further electronic states below our energy cut-off. Hence, it is apparent that our model, while complex for a variational model of its kind, is an empirical description of a much larger problem. Consequently, the empirical nature of the spin-orbit couplings used in this model could lead to erroneous intensity borrowing for transitions arising from energy levels outside of the empirical marvel data set.

**Figure 4.**
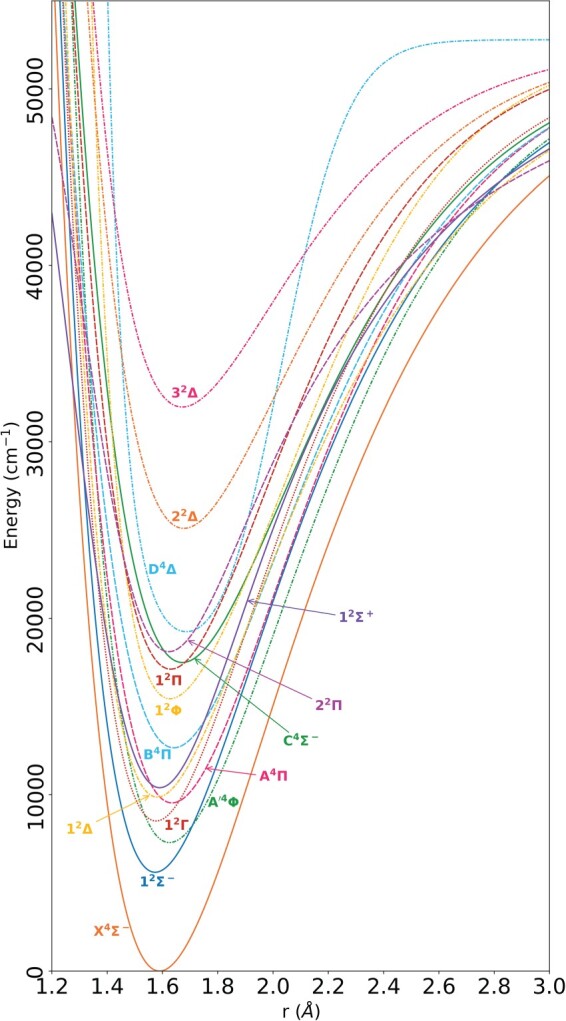
The 15 potential energy curves of 51V 16O included in this model, after refinement against empirical marvel energy levels. Calculations were performedover an internuclear distance range of 1.2 to 4.0 Å; all states tend to the same dissociation asymptote.

Couplings to the D 4Δ state by some of the other electronic states not considered here could explain why it was necessary to fit a much steeper potential for this state in our model, as can be seen in Figure 4. This explanation is perhaps incomplete however, as the *ab initio* calculations of Hübner *et al.* [15] and later calculations by others using the same methodology [86] found the C 4Σ− state had a higher Te than the D 4Δ state and instead adiabatically correlated to the O( 3P) + V(3d 44s 1, 6D) asymptote. Given their state ordering is not in agreement with experimental data, we retained the original O( 3P) + V(3d 34s 2, 4F) dissociation channel for the C 4Σ− state as calculated by McKemmish *et al.* [17], which is the same as all other states in our model.

Under hyperfine coupling, the ΔJ=0,±1 dipole selection rule transforms to ΔF=0,±1, which given the magnitude of the nuclear spin of the 51V atom, facilitates transitions up to ΔJ=±8. While earlier work has shown that these high ΔJ transitions are weak, those with |Δ|J≤3 have intensities on the same order as standard ΔJ=0,±1 transitions [65]. The mean hyperfine splittings of |Δ|J≤3 transitions in the B 4Π–X 4Σ− and C 4Σ−–X 4Σ− (0,0) bands in the region for which marvel energies are known are approximately 0.198 and 0.188 cm −1 respectively, though regularly exceed 0.4 cm −1. In these two bands at roughly 12 600 cm −1 and 17 400 cm −1, high-resolution observations such as those using cross-correlation spectroscopy will aim to resolve separations of 0.126 and 0.174 cm −1 respectively. Hence, hyperfine splittings of VO should be resolvable in high-resolution spectroscopy in the visible and near-infrared regions. Moreover, the overall profile formed by the convolution of individual hyperfine components' line profiles is significantly different from that of a hyperfine-unresolved line profile [65]. Hence, the inclusion of the hyperfine couplings presented here has been done to facilitate the creation of a new line list for VO that can reliably lead to detections with high-resolution observation.

## 5. Conclusions

A hyperfine-resolved spectroscopic model that well-describes 15 electronic states of 51V 16O has been computed using the variational nuclear motion programme Duo and is a key step in the subsequent creation of a new molecular line list. Though the model is imperfect in that it does not exactly recreate experimental data, subsequent work will be undertaken to marvelise this model (see [87,88] for example) for use in high-resolution studies [89]. The model presented here is unusually complex for a variational model, in terms of the number of potential energy and coupling curves involved, though such complexity is necessary to tackle the long-standing problem of VO observations in exoplanet atmospheres [45,47,90–101].

Though the quality of fit to the VO marvel data shown in the hyperfine-unresolved, 11 electronic state model of Qu *et al.* [66] is superior to the fits presented here in some vibronic bands, their model only included two off-diagonal spin-orbit coupling curves. The inclusion of 28 off-diagonal spin-orbit couplings in this work, as well as the new hyperfine couplings, significantly increases the degree of correlation between any given parameters in the model. Accordingly, it took a long time to achieve the fit presented here. For comparison, the quality of the fit presented here is on the same order as those achieved for line lists involving other heavy metal oxides such as CaO [61], TiO [102], ZrO [103] and YO [104]. None of these, however, involved as many electronic states and spin-orbit couplings and crucially, none considered hyperfine effects; indeed 40Ca, 48Ti and 90Zr, the most abundant isotope in each case, are all spin zero nuclei.

While only four states have published hyperfine-resolved spectra, Merer *et al.* [20] observed broadening due to hyperfine splittings in the A ′4Φ–D 4Δ band, but were unable to assign them due to limited experimental resolution. Additional hyperfine-resolved observations of such bands would allow for the inclusion of hyperfine couplings for additional electronic states. Observations of higher vibrational bands would also allow for better constraints on the shapes of the electronic potentials and hence better extrapolation to higher energies. The model presented here covers all of the experimentally observed states of VO, except for the 3 2Π state observed by Hopkins *et al.* [23]. Assignments to the 3 2Π–X 4Σ− bands observed by them could be added to the marvel list and our current model subsequently extended to include the 3 2Π state. Given the considerable computer resources required to compute this model however, the inclusion of additional electronic states and associated couplings may prove challenging without further optimisations to the code.

## Supplementary Material

Supplemental Material

## Data Availability

The Duo input file used to compute the new model and the MARVEL transitions and energy levels used for refining the model are given as supporting material. The Duo input file contains descriptions of all potential energy and coupling curves in the model and can be used to recreate the figures presented here. The Duo code is freely available at https://github.com/Trovemaster/Duo. The version used in this work was compiled from commit e88cbb4 on May 25 2023.
